# Correlates and Predictors of COVID-19 Vaccine Hesitancy Among Persons Living With HIV in Trinidad and Tobago

**DOI:** 10.7759/cureus.35961

**Published:** 2023-03-09

**Authors:** Nyla Lyons, Brendon Bhagwandeen, Selena Todd, Gregory Boyce, Wendy Samaroo-Francis, Jeffrey Edwards

**Affiliations:** 1 Health Research Department, Medical Research Foundation of Trinidad and Tobago, Port of Spain, TTO; 2 School of Mathematical and Computer Sciences, Heriot-Watt University Malaysia, Putrajaya, MYS; 3 Para-Clinical Sciences Department, The University of the West Indies, St. Augustine, TTO

**Keywords:** vaccine willingness, vaccine hesitancy, trinidad and tobago, hiv, covid-19

## Abstract

Background: Persons living with HIV may be at risk of more severe forms of COVID-19 infection and minimizing health risks largely depends on their acceptance of the COVID-19 vaccinations.

Objective: This study examined the correlates and predictors of COVID-19 vaccine hesitancy among persons living with HIV in Trinidad and Tobago.

Methods: A cross-sectional survey using a structured interview was conducted. Data were compiled on patient socio-demographics, diagnosed chronic diseases, psychological factors, and decisions to take the COVID-19 vaccine. Pearson χ^2^ tests examined the associations between study variables and COVID-19 vaccine hesitancy, and multivariable logistic regression analyses examined its predictors.

Results: In this study, 84% were virally suppressed, i.e., HIV viral load <1000 copies/ml. COVID-19 vaccine hesitancy was found to be 39%. Univariate analysis showed that higher vaccine hesitancy was significantly associated with females (OR 2.02, 95% CI 1.23-3.33) and patients of mixed ethnicity (OR 1.84, 95% CI 1.07-3.15). In our multivariable analysis, psychological factors namely, confidence in the COVID-19 vaccine (OR 0.16, 95% CI 0.05-0.47), the perceived benefits of the vaccine (OR 0.54, 95% CI 0.37-0.79), and cues to action (OR 0.68, 95% CI 0.47-0.97) were observed as predictors of COVID-19 vaccine hesitancy.

Conclusion: Psychological factors such as confidence in the COVID-19 vaccine, perceived benefits of the vaccine, and cues to action were possible predictors of COVID-19 vaccine hesitancy. This study underscored the continued need for strategies to increase confidence and knowledge about the benefits of taking the COVID-19 vaccine among persons living with HIV.

## Introduction

In the Caribbean, the twin island republic of Trinidad and Tobago has an estimated population of 1.39 million persons (2021 mid-year estimate) with the ethnic composition comprising 35.4% of East Indian origin, 34.2% of African ancestry, 23% of mixed races, and 8.4% of other ethnic groups (including European, Asian, Middle Eastern) [1]. The first confirmed case of the COVID-19 virus in Trinidad and Tobago was reported on March 12, 2020, and by the end of May 2022, over 161,000 positive cases were reported. This represented approximately 12% of the total population. The country began its COVID-19 vaccination program in April 2021 with mass vaccination sites situated across the country and extensive education campaigns to strengthen the public’s perception and confidence in the safety and efficacy of COVID-19 vaccinations [2]. In May 2022, the Ministry of Health of Trinidad and Tobago reported that 59.8% of the population was fully vaccinated [2].

The World Health Organization (WHO) defined vaccine hesitancy as a behavior influenced by several factors including issues of confidence, i.e., mistrust in the vaccine or healthcare provider; complacency, i.e., a low perceived need and value attached to the vaccine; and convenience, i.e., access and availability of vaccines [3]. Vaccine-hesitant individuals are a heterogeneous group who hold varying degrees of indecision about specific vaccines or vaccination in general. Vaccine-hesitant individuals may accept all vaccines but remain concerned about them; some may refuse or delay some vaccines but accept others, while some individuals may refuse all vaccines [3]. Vaccine hesitancy may also be affected by factors that include cognitive, psychological, socio-demographic, political, and cultural components, which may differ across populations and subpopulations. In the context of the COVID-19 vaccine, the historic speed with which the vaccine was developed facilitated a storm of misinformation from social and alternative media, thus eroding vaccine confidence and contributing to vaccine hesitancy [4].

Beginning in mid-2020, several cross-sectional studies were conducted examining COVID-19 vaccine willingness and hesitancy in the general population in Trinidad and Tobago. In the last quarter of 2020, one of the first studies which included Trinidad and Tobago was conducted by the Johns Hopkins Centre for Communication Programs, which reported that 59% of persons were COVID-19 vaccine-hesitant [5]. At the end of 2020, another survey examined willingness to receive the COVID-19 vaccine and found that 62.8% indicated that they would take the COVID-19 vaccine. The study also observed that lower levels of health literacy were significantly associated with higher levels of misinformation about the vaccine. However, persons reported that the two most trusted sources of information about the COVID-19 vaccines were health workers (32.5%) and the Ministry of Health (23.6%) [6]. Following the public availability of COVID-19 vaccines in April 2021, a survey was conducted by Market Facts and Opinions (MFO) which investigated the public’s opinions about COVID-19 vaccines. It was observed that 65% of persons were unwilling to accept the vaccine [7]. UNICEF later reported that of the persons surveyed in Trinidad and Tobago, 65% were vaccinated against the COVID-19 virus. Vaccine safety and efficacy were among the main reasons for persons’ decisions to not accept the COVID-19 vaccine [8].

While the generalizability of these cross-sectional studies was limited, the prevalence of vaccine hesitancy on average was found to be approximately 60% among adults in the general population [4]. Vaccine hesitancy has been recognized as an ongoing challenge to achieving national vaccination coverage, controlling the spread of the COVID-19 virus, and reducing COVID-19 disease morbidity and mortality.

The WHO recommended that persons living with HIV (PLHIV) should be prioritized for early COVID-19 vaccinations as HIV infection was reported to be a significant independent risk factor for severe COVID-19 presentation on hospital admission, i.e., PLHIV were hospitalized at higher rates and presented a higher mortality risk than non-PLHIV [3]. Findings from a large population-based cohort in South Africa showed the COVID-19 mortality risk among people living with HIV was double the risk of those without HIV [9]. Furthermore, in China, HIV-infected patients with COVID-19 presented a higher prevalence of critical illnesses compared to those without HIV [10]. In the United Kingdom (UK), HIV patients had a higher risk of COVID-19 death than those without HIV [9,11]. Given their increased risk for health consequences, the main objective of our study was to examine the correlates and predictors of COVID-19 vaccine hesitancy among patients with HIV. This work has not been previously undertaken in Trinidad and Tobago and examining factors associated with COVID-19 vaccine hesitancy among patients with HIV could be vital to inform the implementation of strategies to reduce COVID-19 vaccine hesitancy and increase vaccine uptake among this high-risk population.

## Materials and methods

Study design and participants

This cross-sectional survey was implemented in August 2021, targeting PLHIV in Trinidad and Tobago, through structured face-to-face interviews. The Medical Research Foundation of Trinidad and Tobago (MRFTT) facilitated the study. The MRFTT is recognized as the largest HIV Treatment and Care Centre in Trinidad and Tobago. Adhering to the national COVID-19 guidelines, the MRFTT implemented procedures to alleviate crowded waiting rooms. Therefore, patient appointments fell considerably each month such that approximately 800 patients comprised the available target population for the duration of the study. Using a margin of error of 5%, a confidence level of 95%, and a 60% vaccine hesitancy rate based on the average reported in the general population, this gave a minimum sample size of n = 253 participants. The final sample comprised n = 272 patients meeting the eligibility criteria and giving their consent to participate in the study.

The eligibility criteria included PLHIV in Trinidad and Tobago, aged 18 years and older. These persons were provided with details about the study and participated after their written consent was provided. Nurses at the MRFTT were trained in data collection and given the inclusion criteria for the study. Participant confidentiality was maintained throughout the survey. Physical copies of the completed questionnaires were secured at the MRFTT, and the resulting database was stored on a password-protected computer, with only members of the research team having access to this data.

Study questionnaire and data collection

A structured questionnaire was used during interviews and consisted of the following sections: (1) sociodemographic characteristics; (2) presence of chronic diseases; (3) self-rated health status; (4) exposure to COVID-19; (5) COVID-19 vaccination hesitancy; (6) psychological factors; (7) length of time since HIV diagnosis; and (8) viral suppression. Each interview took approximately 15 minutes to complete. Table 1 provided further details of the study variables.

**Table 1:** Variables and measurement

Variable	Measurement
Sociodemographic characteristics	Age, gender, ethnicity, marital status, education level, employment status and sexual orientation
COVID-19 vaccine hesitancy	If offered the COVID-19 vaccine, would you take it?
Psychological factors	Measurement
Confidence in the COVID-19 vaccine	I am confident about the COVID-19 vaccines
Perceived susceptibility of contracting COVID-19	1. My chance of getting COVID-19 is great.
	2. I am worried about the likelihood of getting COVID-19.
	3. My present health condition makes it easier for me to contract COVID-19 infection.
	4. The medications I am currently taking can protect me from contracting COVID-19 infections.
Perceived severity of COVID-19	1. Complications from COVID-19 vaccination are serious.
	2. I will be very sick if I take the COVID-19 vaccine.
	3. I am afraid of taking the COVID-19 vaccine.
Perceived benefits of the COVID-19 vaccine	1. Vaccination is a good idea because I feel less worried about catching COVID-19.
	2. Vaccination decreases my chance of getting COVID-19 or its complications.
Perceived concerns about the COVID-19 vaccine	1. I am worried the possible side effects of COVID-19 vaccination would interfere with my usual activities.
	2. I am concerned about the effectiveness of the COVID-19 vaccination.
	3. I am concerned about the safety of the COVID-19 vaccination.
	4. I am concerned that the COVID-19 vaccine needs more testing before I feel safe to take it.
	5. I am concerned of the faulty/fake COVID-19 vaccine.
Cues to action	1. I will only take the COVID-19 vaccine if I was given adequate information about it.
	2. I will only take the COVID-19 vaccine if the vaccine is taken by many in the public.

Psychological factors were scored on a 4-point Likert scale, with a score of one (1) indicating strong disagreement and a score of four (4) indicating strong agreement. Item 4: “The medications I am currently taking can protect me from contracting COVID-19 infections”, of the perceived susceptibility scale was reverse coded to maintain internal consistency. An overall score was then created to measure each construct using the sum of individual scores of the responses from the Likert scales. COVID-19 vaccine hesitancy was a dichotomous variable such that “0 = not hesitant to take the COVID-19 vaccine" and “1 = hesitant to take the COVID-19 vaccine”.

Reliability of psychological constructs

The questionnaire was pre-tested. Medical doctors and medical research experts at the MRFTT also reviewed the questionnaire design, content, words, comprehension, and ease of completion.

The Cronbach α coefficients for the psychological components showed low to high internal consistency. The reliability statistics were presented in Table 2. All psychological measurements were retained since medical professionals and public health researchers at the MRFTT believed that understanding these perceptions was vital and relevant in the context of the COVID-19 pandemic.

**Table 2:** Internal consistency of psychological measurements of the study questionnaire

Psychological measurements	Cronbach α coefficient
Perceived susceptibility to COVID-19	0.55
Cues to action	0.58
Perceived severity of COVID-19	0.78
Perceived benefits of COVID-19 vaccination	0.86
Perceived barriers about COVID-19 vaccination	0.88

Statistical analysis

All data were cleaned, coded, and analyzed using Statistical Product and Service Solutions (SPSS) (IBM SPSS Statistics for Windows, Version 22.0, Armonk, NY) [12]. Descriptive statistics were presented as mean ± standard deviation (SD) for continuous variables and frequencies with percentages for categorical variables. These were used to characterize baseline distributions of study variables. Differences were compared using independent samples t-tests for continuous variables and Pearson χ^2^ tests for categorical variables with Bonferroni correction for multiple hypothesis testing. Logistic regression was used to examine unadjusted and adjusted odds ratios (OR) with 95% confidence intervals (CI) for associations between COVID-19 vaccine hesitancy with psychological factors and sociodemographic characteristics. Psychological measures were used as possible predictors of COVID-19 vaccine hesitancy while controlling for sociodemographic confounders in the multivariable regression analysis. Analyses included only non-missing data. Statistical significance was set at p < 0.05.

Ethical approval

The study protocol, data collection instruments, and informed consent form were approved by the Campus Research Ethics Committee of the University of the West Indies, St. Augustine, Trinidad, approval number CREC-SA.1063/06/2021.

## Results

Characteristics of participants living with HIV

Table 3 summarized the sociodemographic characteristics of participants living with HIV in this study. A total of 272 individuals at an HIV treatment clinic in Trinidad and Tobago completed the survey. Gender was evenly distributed with 135 (49.6%) males and 137 (50.4%) females. Most participants were of African origin (n = 161, 59.2%), single (n = 179, 65.8%), received up to secondary schooling (n = 172, 63.2%), employed (n = 132, 50.4%), and heterosexual (n = 192, 70.6%). The average age of participants in the study was reported to be 41.7 ± 13.0 years.

**Table 3:** Sociodemographic characteristics of participants

Variables	n	%
Gender		
Male	135	49.6
Female	137	50.4
Total	272	100.0
Ethnicity		
African origin	161	59.2
East Indian origin	25	9.2
Mixed/Other	84	30.9
Non-responder	2	0.7
Total	272	100.0
Marital status		
Married	28	10.3
Single	179	65.8
Common law/Other	65	23.9
Total	272	100.0
Education level		
Primary school or below	41	15.1
Secondary school	172	63.2
University or above	58	21.3
Non-responder	1	0.4
Total	272	100.0
Employment status		
Employed	137	50.4
Unemployed	107	39.3
Other	28	10.3
Total	272	100.0
Sexual orientation		
Heterosexual	192	70.6
Non-heterosexual	68	25.0
Non-responder	12	4.4
Total	272	100.0
Previous or current exposure to the COVID-19 virus		
No	249	91.5
Yes	19	7.0
No-response	4	1.5
Total	272	100.0
Family/friends contracting COVID-19		
No	202	74.3
Yes	70	25.7
Total	272	100.0
Existing chronic diseases		
No	195	71.7
Yes	73	26.8
Non-responder	4	1.5
Total	272	100.0
Self-rated health status		
Poor health	55	20.2
Good health	217	79.8
Total	272	100.0
Confidence in the COVID-19 vaccine		
Not confident	139	51.1
Confident	129	47.4
Non-responder	4	1.5
Total	272	100.0
Viral suppression (VL<1000 copies/ml)		
Not suppressed	39	14.3
Suppressed	229	84.2
Total	272	100.0
COVID-19 vaccine hesitancy		
Not hesitant	163	59.9
Hesitant	106	39.0
Non-responder	3	1.1
Total	272	100.0
	Mean ± SD	
Age (years)	41.7 ± 13.0	
Length of time since HIV diagnosis and initiation on ART (years)	8.73 ± 6.02	

Most of the participants (n = 249, 91.5%) reported not being previously exposed to COVID-19. More than one-quarter of the patients (n = 73, 26.8%) reported having been diagnosed with chronic diseases, and almost 80% rated themselves as being in good health.

Almost half of the participants (n = 129, 47.4%) indicated that they were confident in the COVID-19 vaccine. Overall, 106 patients (39.0%) were hesitant to take the COVID-19 vaccine. Most PLHIV (n = 229, 84%) were virally suppressed, with patients being initiated on antiretroviral therapy (ART) for an average of 8.73 ± 6.02 years since the time of initial diagnosis.

Differences in COVID-19 vaccine hesitancy and sociodemographic characteristics

Table 4 presented the differences in COVID-19 vaccine hesitancy when considering sociodemographic characteristics. Significant differences in COVID-19 vaccination hesitancy were observed for ethnicity (p = 0.001) and confidence in the COVID-19 vaccine (p < 0.001) based on Bonferroni-adjusted p-values for significance (p < 0.004). Persons of East Indian descent (12%) had the lowest proportion for individuals who were hesitant to get vaccinated, followed by persons of African origin (37.5%) and persons of mixed/other ethnicities (52.4%). The proportion of persons who were hesitant to get vaccinated given that they were confident in the COVID-19 vaccine (8.7%) was much lower than the proportion of persons who were hesitant to get vaccinated but were not confident in it (68.3%).

**Table 4:** Differences in COVID-19 vaccine hesitancy according to sociodemographic characteristics, row %

Variable	COVID-19 vaccine hesitancy		
	Not hesitant	Hesitant	p-value
Gender			0.005
Male	93 (68.9%)	42 (31.1%)	
Female	70 (52.2%)	64 (47.8%)	
Ethnicity			0.001
Afro-Trinidadian	100 (62.5%)	60 (37.5%)	
Indo-Trinidadian	22 (88.0%)	3 (12.0%)	
Mixed	39 (47.6%)	43 (52.4%)	
Marital status			0.523
Married	15(53.6%)	13 (46.4%)	
Single	112 (62.9%)	66 (37.1%)	
Common law/Other	36 (57.1%)	27 (42.9%)	
Education level			0.224
Primary school or below	28 (70.0%)	12 (30.0%)	
Secondary school	97 (56.7%)	74 (43.3%)	
University or above	37 (64.9%)	20 (35.1%)	
Employment status			0.164
Employed	90 (66.2%)	46 (33.8%)	
Unemployed	58 (55.2%)	47 (44.8%)	
Other	15 (53.6%)	13 (46.4%)	
Sexual orientation			0.047
Heterosexual	110 (57.9%)	80 (42.1%)	
Non-heterosexual	48 (71.6%)	19 (28.4%)	
Previous or current exposure to COVID-19 disease			0.824
No	149 (60.6%)	97 (39.4%)	
Yes	12 (63.2%)	7 (36.8%)	
Family/friends contracting COVID-19			0.138
No	116 (58.0%)	84 (42.0%)	
Yes	47 (68.1%)	22 (31.9%)	
Existing chronic diseases			0.560
No	118 (61.5%)	74 (38.5%)	
Yes	42 (57.5%)	31 (42.5%)	
Self-rated health status			0.919
Poor health	33 (60.0%)	22 (40.0%)	
Good health	130 (60.7%)	84 (39.3%)	
Confidence in the COVID-19 vaccine			< 0.001
Not confident	44 (31.7%)	95 (68.3%)	
Confident	116 (91.3%)	11 (8.7%)	
Viral suppression			0.955
Not suppressed	24 (61.5%)	15 (38.5%)	
Suppressed	138 (61.1%)	88 (38.9%)	
Age	42.3 ± 13.0	40.6 ± 13.2	0.304
Length of time since HIV diagnosis and initiation on ART	8.47 ± 6.04	8.99 ± 5.98	0.486

No statistically significant differences in COVID-19 vaccination hesitancy were reported for gender (p = 0.005), marital status (p = 0.523), education (p = 0.224), employment status (p = 0.164), sexual orientation (p = 0.047), prior experience with COVID-19 (p = 0.824), whether family/friends contracted COVID-19 (p = 0.138), presence of chronic diseases (p = 0.560), self-rated health status (p = 0.919) and viral suppression (p = 0.955) based on Bonferroni-adjusted p-values for significance. There were also no significant differences when age (p = 0.304) and length of time since HIV diagnosis and initiation on ART (p = 0.486) were used as independent variables.

Associations between COVID-19 vaccine hesitancy and sociodemographic characteristics

Table 5 presented univariate associations between COVID-19 vaccine hesitancy and sociodemographic characteristics. Females had twice the odds (OR 2.02, 95% CI 1.23-3.33) of being hesitant to take the COVID-19 vaccine relative to males, while East Indians had lower odds (OR 0.23, 95% CI 0.07-0.79) of being hesitant to take the COVID-19 vaccine, but persons of mixed ethnicity had greater odds (OR 1.84, 95% CI 1.07-3.5) of being hesitant, relative to persons of African descent. Persons who were confident in the COVID-19 vaccine had considerably lower odds (OR 0.04, 95% CI 0.02-0.09) of being hesitant to take the COVID-19 vaccine relative to persons who were not confident.

**Table 5:** Unadjusted odds ratios (with 95% CI) for COVID-19 vaccine hesitancy and sociodemographic characteristics OR: odds ratio; CI: confidence interval

Variable	COVID-19 vaccine hesitancy (Ref: Not hesitant)		
	Unadjusted OR	95% CI	p-value
Gender			
Male	Ref	Ref	
Female	2.02	1.23-3.33	0.005
Ethnicity			
Afro-Trinidadian	Ref	Ref	
Indo-Trinidadian	0.23	0.07-0.79	0.020
Mixed	1.84	1.07-3.15	0.027
Marital status			
Married	Ref	Ref	
Single	0.68	0.31-1.52	0.346
Common law/Other	0.87	0.35-2.12	0.751
Education level			
Primary school or below	Ref	Ref	
Secondary school	1.78	0.85-3.73	0.127
University or above	1.26	0.53-3.00	0.600
Employment status			
Employed	Ref	Ref	
Unemployed	1.59	0.94-2.68	0.084
Other	1.70	0.74-3.86	0.209
Sexual orientation			
Heterosexual	Ref	Ref	
Non-heterosexual	0.54	0.30-1.00	0.048
Previous or current exposure to COVID-19 disease			
No	Ref	Ref	
Yes	0.90	0.34-2.36	0.824
Family/friends contracting COVID-19			
No	Ref	Ref	
Yes	0.65	0.36-1.15	0.140
Existing chronic diseases			
No	Ref	Ref	
Yes	0.56	0.68-2.04	0.560
Self-rated health status			
Poor health	Ref	Ref	
Good health	0.97	0.53-1.78	0.919
Confidence in the COVID-19 vaccine			
Not confident	Ref	Ref	
Confident	0.04	0.02-0.09	< 0.001
Viral suppression			
Not suppressed	Ref	Ref	
Suppressed	1.02	0.51-2.05	0.955
Age	0.99	0.97-1.01	0.303
Length of time since HIV diagnosis and initiation on ART	1.02	0.97-1.06	0.484

There were no significant associations between COVID-19 vaccine hesitancy with marital status, education level, employment status, experience with COVID-19, family/friends contracting COVID-19, existing chronic diseases, self-rated health status, viral suppression, age, or length of time since HIV diagnosis and initiation on ART and COVID-19 vaccine hesitancy.

COVID-19 vaccine hesitancy and psychological factors

Table 6 presented COVID-19 vaccine hesitancy among PLHIV in Trinidad and Tobago based on the psychological factors considered in this study. Three of the five psychological factors were found to be significantly associated with COVID-19 vaccine hesitancy based on Bonferroni adjusted p-values needed for significance (p < 0.01). Higher scores on the perceived severity of the COVID-19 scale were associated with COVID-19 vaccine hesitancy (not hesitant: 6.82 ± 1.88, hesitant: 8.95 ± 2.00; p < 0.001). Lower scores on the perceived benefits of the COVID-19 vaccine scale were associated with COVID-19 vaccine hesitancy (not hesitant: 6.01 ± 1.22, hesitant: 4.24 ± 1.40; p < 0.001). Higher scores on the perceived concerns of the COVID-19 vaccine scale were associated with COVID-19 vaccine hesitancy (not hesitant: 12.6 ± 3.37, hesitant: 16.0 ± 3.03; p < 0.001).

**Table 6:** COVID-19 vaccine hesitancy and COVID-19 vaccine psychological measures

Psychological construct	Mean score ± SD	COVID-19 vaccine hesitancy		
		Not hesitant	Hesitant	p-value
Perceived susceptibility of contracting COVID-19	10.2 ± 2.18	10.5 ± 2.09	9.74 ± 2.27	0.011
Perceived severity of COVID-19	7.65 ± 2.19	6.82 ± 1.88	8.95 ± 2.00	< 0.001
Perceived benefits of the COVID-19 vaccine	5.32 ± 1.56	6.01 ± 1.22	4.24 ± 1.40	< 0.001
Perceived barriers of the COVID-19 vaccine	13.9 ± 3.64	12.6 ± 3.37	16.0 ± 3.03	< 0.001
Cues to action	5.46 ± 1.37	5.63 ± 1.25	5.18 ± 1.52	0.014

Predictors of COVID-19 vaccine hesitancy in persons living with HIV

Table 7 presented the results of our multivariable logistic regression analysis for COVID-19 vaccine hesitancy. Patient’s confidence in the COVID-19 vaccine was observed as a significant predictor in this study, with the odds of vaccine hesitancy being lower among persons who were confident in the COVID-19 vaccine (OR 0.16, 95% CI 0.05-0.47) relative to persons with persons who were not confident. The perceived benefits of the COVID-19 vaccine (OR 0.54, 95% CI 0.37-0.79) and cues to action (OR 0.68, 95% CI 0.47-0.97) measures were also observed to be potential predictors of COVID-19 vaccine hesitancy among PLHIV attending the treatment clinic in Trinidad and Tobago.

**Table 7:** Adjusted odds ratios (with 95% CI) for COVID-19 vaccine hesitancy with psychological measures and sociodemographic characteristics OR: odds ratio; CI: confidence interval

Variable	COVID-19 vaccine hesitancy (Ref: Not hesitant)		
	Adjusted OR	95% CI	p-value
Gender			
Male	Ref	Ref	
Female	0.68	0.28-1.66	0.400
Ethnicity			
Afro-Trinidadian	Ref	Ref	
Indo-Trinidadian	0.40	0.09-1.81	0.234
Mixed	2.41	0.96-6.02	0.061
Sexual orientation			
Heterosexual	Ref	Ref	
Non-heterosexual	0.43	0.15-1.28	0.130
Confidence in the COVID-19 vaccine			
No	Ref	Ref	
Yes	0.16	0.05-0.47	0.001
Perceived susceptibility of contracting COVID-19	0.96	0.77-1.20	0.711
Perceived severity of COVID-19	1.34	0.96-1.87	0.090
Perceived benefits of the COVID-19 vaccine	0.54	0.37-0.79	0.001
Perceived concerns/ barriers of the COVID-19 vaccine	1.03	0.84-1.26	0.793
Cues to action	0.68	0.47-0.97	0.029

Gender, ethnicity, sexual orientation and the perceived susceptibility of contracting COVID-19, the perceived severity of COVID-19, and perceived concerns/barriers of the COVID-19 vaccine constructs were not found to be significant predictors of COVID-19 vaccine hesitancy in this study.

## Discussion

The objective of our study was to examine the correlates and predictors of COVID-19 vaccine hesitancy among patients with HIV in Trinidad and Tobago. The study included patients’ sociodemographic characteristics, existing chronic diseases, self-rated health status, COVID-19 experience, and psychological factors.

Our results showed that COVID-19 vaccine hesitancy was 39% among PLHIV in this sample, which was lower than in studies conducted among persons in the general population (i.e., vaccine hesitancy was estimated between 59% and 65% in the general population) [4,7]. These results were consistent with previous research findings, reporting vaccine hesitancy rates between 27.5% and 38.4% among PLHIV across various settings [10,13].

In this sample, 84% of the patients were virally suppressed (VL <100 copies/ml), demonstrating high levels of treatment success. Over 75% of our sample reported having no experience with the COVID-19 virus, i.e., had not been clinically diagnosed with COVID-19. Over 75% also reported that they were in good health, with no diagnosed chronic diseases.

Since the first confirmed case of the COVID-19 virus in Trinidad and Tobago in March 2020, the MRFTT implemented several measures such as six-month ART dispensing, telehealth, and community ART pick-up, reducing barriers to care during the COVID-19 pandemic and ensuring patients maintained their medication regimens and remained virally suppressed. These strategies may have contributed to the high levels of viral suppression among patients in our sample. This study was also implemented six months after the COVID-19 vaccine was made available to the public (i.e., April 2021), during which time, information on the consequences of the COVID-19 virus and on the vaccine’s safety and efficacy for use among PLHIV were still evolving. This may have contributed to the patient’s hesitancy to take the vaccine. Therefore, it was important for HIV treatment facilities like the MRFTT to continue to implement educational campaigns tailored to the specific concerns of PLHIV and COVID-19 vaccination.

Based on our univariate analysis, among the sociodemographic characteristics in our study, females had twice the odds of being vaccine-hesitant compared to males and patients of mixed ethnicity also had greater odds of being hesitant compared to patients of African descent. Patients identifying as non-heterosexuals (i.e., gay and lesbian) had lower odds of being hesitant when compared to heterosexuals. These study findings were consistent with research on COVID-19 vaccine hesitancy and the associated demographic factors [14,15]. However, few studies have examined COVID-19 vaccine hesitancy among non-heterosexuals. A multinational systematic review demonstrated that females, membership in an ethnic minority group, and other socio-demographic factors were consistently associated with higher COVID-19 vaccine hesitancy [14,16] and indicated the need for targeted approaches to understand vaccine hesitancy among women and some ethnic minorities to reduce barriers to the uptake of the COVID-19 vaccine. A study implemented in France among MSM (men who have sex with men) found that HIV-positive MSM was less likely to report vaccination uncertainty and unwillingness compared to HIV-negative MSM, and those who were undiagnosed [17]. The results of our study were consistent with these findings of HIV-positive MSM in France, suggesting lower vaccine hesitancy among HIV-positive MSM. Additional research was needed among this subpopulation to better understand factors contributing to vaccine hesitancy among HIV-positive MSM.

Psychological factors related to patients’ beliefs and perceptions about the COVID-19 vaccine were included to investigate the associations with COVID-19 vaccine hesitancy. Our univariate analysis presented significant associations among four of the psychological factors namely, confidence in the COVID-19 vaccines, perceived severity of COVID-19 disease, perceived benefits of the COVID-19 vaccine, and perceived barriers to the COVID-19 vaccine. It was found that lower scores on perceived benefits were associated with higher vaccine hesitancy among patients in our study. Emerging studies supported these findings as persons with a higher level of perception of the benefits of the vaccine had greater odds of being vaccinated [15,18]. Over two-thirds of patients in our sample reported that they were in good health, with no diagnosed commodities, and high levels of viral suppression, and, therefore, might not see themselves at risk for contracting the COVID-19 virus. It was found that higher scores on the perceived severity of the COVID-19 virus were significantly associated with vaccine hesitancy. These findings were consistently supported in previous studies of COVID-19 vaccine hesitancy in the general population, where vaccine-specific concerns (side effects and efficacy) were cited as the most common factors associated with hesitancy [19,20]. Less has been published about a person’s willingness to take the vaccines if given more information about it, and/or knowing many persons who took the vaccine. A study in France showed that emphasizing both the personal benefits such as patient safety concerns about the COVID-19 vaccine and collective benefits such as the collective benefits of herd immunity were important contributors to minimizing vaccine hesitancy among patients with HIV [21].

In this study, there were no significant associations between the presence of diagnosed chronic diseases and COVID-19 vaccine hesitancy. This finding was not consistent with the previous studies which found that patients with underlying comorbid disease conditions were more likely to take the COVID-19 vaccine than those without underlying conditions [22,23]. The difference might be due to high levels of viral suppression among patients in our study - many of whom indicated good health and, therefore, having an existing health condition did not affect how they felt about receiving a vaccine at the time.

In the multivariable analysis, patients’ confidence in the COVID-19 vaccine, the perceived benefits of the COVID-19 vaccine, and cues to action were observed as possible predictors of COVID-19 vaccine hesitancy. Over the course of the pandemic, there was early evidence of the storm of misinformation that developed around COVID-19, largely from social and alternative media sources, and coupled with mistrust of government and other authority figures, eroded confidence in COVID-19 containment measures and contributed to vaccine hesitancy [24].

COVID-19 vaccine hesitancy might have also resulted from skepticism about the reliability and competence of the health service system, and mistrust of healthcare providers [25,26]. In a longitudinal study of HIIV-positive African-American men, medical mistrust was found to be associated with lower medication adherence and virology failure [27]. Studies of COVID-19-related mistrust, among HIV-positive African Americans, found that mistrust of the COVID-19 vaccine was associated with higher COVID-19 vaccine hesitancy [28]. While factors related to reliability and trust in the health system and its professionals were not measured in this study, these might have contributed to patients’ confidence about the COVID-19 vaccine and hence their decision not to take the vaccine. In a single, blind randomized controlled trial of adults in the UK, persons who were hesitant to take the COVID-19 vaccine were patients who had little knowledge of the public health benefits of vaccinations or would have considerable doubts about the efficacy of the vaccine and were worried about potential side-effects and issues related to vaccine safety [29]. In a survey of 19 countries to determine potential acceptance rates and factors influencing acceptance of the COVID-19 vaccines, respondents who reported higher levels of trust in information from government sources were more likely to accept a vaccine and take their employer’s advice to do so [30]. The relationship between the perceived benefits of the COVID-19 vaccines and vaccine hesitancy was supported in previous research. In a study using a nationally representative sample to investigate how persons decided to take a COVID-19 vaccine, it was observed that the main difference between hesitant participants and those accepting the vaccine was how they comprehended the benefits of the vaccine and the substantial gap in knowledge among the hesitant, compared with accepting participants [31]. In another study to evaluate the perceptions and hesitancy towards the COVID-19 vaccine, it was found that of the 1287 participants, just over half (n = 665, 52%) reported not having adequate information on the benefits of COVID-19 vaccination [32]. Less has been published about “cues to action” such as a person’s willingness to take the vaccines if given more information about it, and/or knowing many persons who took the vaccine.

Overall, the results of this study highlighted the importance of boosting confidence in the COVID-19 vaccines' safety and effectiveness. In the context of PLHIV, it was critical to provide tailored messages and implement strategies to reinforce both the personal and the social/collective benefits of getting vaccinated against the COVID-19 virus. Strategies to build vaccine literacy and acceptance, addressing the specific concerns of PLHIV and their knowledge of the vaccine’s benefits and the reliability of the healthcare system were required. Strategies should also be implemented to build vaccine literacy and dispel misconceptions, especially as they related to PLHIV.

With the announcement of the availability of COVID-19 vaccinations to the public, clinicians at the MRFTT (including nurses, doctors, and social workers) began educating and counseling patients about COVID-19 vaccinations during their visits to the MRFTT. Information about the benefits of the vaccine was further reinforced through the distribution of information brochures specifically addressing the patients' concerns about the potential for drug interactions between the COVID-19 vaccine and their HIV treatment regimens, and the overall safety of PLHIV. Information about the benefits of the COVID-19 vaccinations was also shared via a dedicated social media page and on television displays at the clinic during the daytime and evening clinic hours. Targeted messages to patients were further provided using peer-to-peer educators who served as advocates to reduce COVID-19 vaccine hesitancy among patients. An important role for patient peers was to provide personal testimonials about their experience with the COVID-19 virus and/or on their receipt of the COVID-19 vaccination. Patients' peers also provided group counseling to further dispel the myths and misconceptions around the COVID-19 vaccinations, side-effects, reduce stigma and alleviate patients' fears about contracting the virus. In October 2021 (following the completion of the survey), the MRFTT became a designated COVID-19 vaccination site approved by the Ministry of Health of Trinidad and Tobago. The intention was to increase access and availability of COVID-19 vaccinations for patients enrolled in care at the MRFTT. COVID-19 vaccinations have since been integrated with the overall vaccine prevention program for patients enrolled in care, who also benefitted from the Flu, Pneumovax, Hepatitis B, and HPV vaccines.

The results of the survey highlighted the need to upscale targeted strategies to increase the uptake of COVID-19 vaccinations among HIV-positive women and among ethnic groups. These results additionally highlighted the need to increase patients' knowledge of the personal and collective benefits of COVID-19 vaccinations such as not spreading the virus to others, and reducing the community spread of the COVID-19 virus.

Study limitations

The study was conducted during the period when COVID-19 vaccines were made available to the public. This corresponded with public mistrust and concerns about the safety and efficacy of the vaccines. During the period of COVID-19 restrictions, several measures were implemented to reduce the potential risk of the spread of COVID-19 infections by patients and staff. Risk reduction interventions such as the use of telehealth, community ART delivery, and six-month ART dispensing substantially reduced the number of in-person visits. Therefore, the study sample may not be representative of the entire PLHIV population in Trinidad and Tobago. Information regarding COVID-19 vaccinations and HIV were still in its early stages during the time of the study, thus contributing to uncertainty over vaccine safety among this subpopulation in Trinidad and Tobago. Unmeasured factors could have also influenced vaccine hesitancy. These included sources of information and trust in the healthcare professionals and the health system. As it pertained to psychological factors, the study did not suggest a strategy for changing health-related actions. Additionally, the cross-sectional nature of the study could potentially leave it susceptible to reverse causality.

## Conclusions

Our study underscored the ongoing need to further develop and continue existing strategies to increase patients’ confidence and knowledge of the benefits of the COVID-19 vaccines since psychological factors including confidence in the COVID-19 vaccine, perceived benefits of the vaccine, and cues to action were observed to be possible predictors of COVID-9 vaccine hesitancy among PLHIV. It remains important for persons to comprehend the psychological factors associated with COVID-19 vaccine hesitancy and facilitate the necessary avenues by which this at-risk subpopulation is provided with the opportunity to share their personal and collective experiences with COVID-19 vaccination.

## References

[1] (2022). Central Statistical Office. Population Statistics Trinidad and Tobago. https://cso.gov.tt/subjects/population-and-vital-statistics/population/.

[2] (2022). Ministry of Health of Trinidad and Tobago. https://health.gov.tt/covid-19/covid-19-news-and-updates.

[3] Troiano G, Nardi A (2021). Vaccine hesitancy in the era of COVID-19. Public Health.

[4] Cascini F, Pantovic A, Al-Ajlouni Y, Failla G, Ricciardi W (2021). Attitudes, acceptance and hesitancy among the general population worldwide to receive the COVID-19 vaccines and their contributing factors: a systematic review. EClinicalMedicine.

[5] Babalola S, Krenn S, Rosen JG (2022). COVID Behaviors Dashboard. Johns Hopkins Center for Communication Programs in collaboration with Facebook Data for Good, Delphi Group at Carnegie Mellon University, University of Maryland Social Data Science Center, Global Outbreak Alert and Response Network. https://ccp.jhu.edu/kap-covid/.

[6] De Freitas L, Basdeo D, Wang HI (2021). Public trust, information sources and vaccine willingness related to the COVID-19 pandemic in Trinidad and Tobago: an online cross-sectional survey. Lancet Reg Health Am.

[7] (2022). Market Facts and Opinions. Key Findings on National Perception of Vaccines. https://www.mfocaribbean.com/mfo-key-findings-on-national-perception-of-vaccines/..

[8] (2022). UNICEF, COVID-19 Vaccine Hesitancy Survey Report. https://www.unicef.org/easterncaribbean/reports/covid-19-vaccine-hesitancy-survey-report-2021.

[9] Jassat W, Cohen C, Tempia S (2021). Risk factors for COVID-19-related in-hospital mortality in a high HIV and tuberculosis prevalence setting in South Africa: a cohort study. Lancet HIV.

[10] Liu Y, Han J, Li X (2021). COVID-19 vaccination in people living with HIV (PLWH) in China: a cross sectional study of vaccine hesitancy, safety, and immunogenicity. Vaccines (Basel).

[11] Geretti AM, Stockdale AJ, Kelly SH (2021). Outcomes of coronavirus disease 2019 (COVID-19) related hospitalization among people with human immunodeficiency virus (HIV) in the ISARIC World Health Organization (WHO) clinical characterization protocol (UK): a prospective observational study. Clin Infect Dis.

[12] IBM Corp (2013). IBM SPSS Statistics for Windows. https://www.ibm.com/spss.

[13] Vallée A, Fourn E, Majerholc C, Touche P, Zucman D (2021). COVID-19 vaccine hesitancy among French people living with HIV. Vaccines (Basel).

[14] Robertson E, Reeve KS, Niedzwiedz CL (2021). Predictors of COVID-19 vaccine hesitancy in the UK household longitudinal study. Brain Behav Immun.

[15] Ng JWJ, Vaithilingam S, Nair M, Hwang LA, Musa KI (2022). Key predictors of COVID-19 vaccine hesitancy in Malaysia: an integrated framework. PLoS One.

[16] Robinson E, Jones A, Lesser I, Daly M (2021). International estimates of intended uptake and refusal of COVID-19 vaccines: a rapid systematic review and meta-analysis of large nationally representative samples. Vaccine.

[17] Ousseine YM, Vaux S, Vandentorren S, Bonmarin I, Champenois K, Lydié N, Velter A (2022). Predictors of uncertainty and unwillingness to receive the COVID-19 vaccine in men who have sex with men in France. Int J Environ Res Public Health.

[18] Guidry JP, Laestadius LI, Vraga EK (2021). Willingness to get the COVID-19 vaccine with and without emergency use authorization. Am J Infect Control.

[19] Orebi HA, Emara HE, Alhindi AA, Shahin MR, Hegazy AH, Kabbash IA, Saied SM (2022). Perceptions and experiences of COVID-19 vaccines' side effects among healthcare workers at an Egyptian University Hospital: a cross-sectional study. Trop Med Health.

[20] Kerr JR, Freeman AL, Marteau TM, van der Linden S (2021). Effect of information about COVID-19 vaccine effectiveness and side effects on behavioural intentions: two online experiments. Vaccines (Basel).

[21] Guillon M, Kergall P (2021). Factors associated with COVID-19 vaccination intentions and attitudes in France. Public Health.

[22] Sanyaolu A, Okorie C, Marinkovic A (2020). Comorbidity and its impact on patients with COVID-19. SN Compr Clin Med.

[23] Mesfin Y, Argaw M, Geze S, Zewdu BT (2021). Factors associated with intention to receive COVID-19 vaccine among HIV positive patients attending ART clinic in Southwest Ethiopia. Patient Prefer Adherence.

[24] Roozenbeek J, Schneider CR, Dryhurst S (2020). Susceptibility to misinformation about COVID-19 around the world. R Soc Open Sci.

[25] Puri N, Coomes EA, Haghbayan H, Gunaratne K (2020). Social media and vaccine hesitancy: new updates for the era of COVID-19 and globalized infectious diseases. Hum Vaccin Immunother.

[26] Jarolimova J, Yan J, Govere S (2021). Medical mistrust and stigma associated with COVID-19 among people living with HIV in South Africa. AIDS Behav.

[27] Dale SK, Bogart LM, Wagner GJ, Galvan FH, Klein DJ (2016). Medical mistrust is related to lower longitudinal medication adherence among African-American males with HIV. J Health Psychol.

[28] Bogart LM, Ojikutu BO, Tyagi K (2021). COVID-19 related medical mistrust, health impacts, and potential vaccine hesitancy among Black Americans living with HIV. J Acquir Immune Defic Syndr.

[29] Freeman D, Loe BS, Yu LM (2021). Effects of different types of written vaccination information on COVID-19 vaccine hesitancy in the UK (OCEANS-III): a single-blind, parallel-group, randomised controlled trial. Lancet Public Health.

[30] Lazarus JV, Ratzan SC, Palayew A (2021). A global survey of potential acceptance of a COVID-19 vaccine. Nat Med.

[31] Robertson DA, Mohr KS, Barjaková M, Lunn PD (2021). A lack of perceived benefits and a gap in knowledge distinguish the vaccine hesitant from vaccine accepting during the COVID-19 pandemic. Psychol Med.

[32] Abu Farha RK, Alzoubi KH, Khabour OF, Alfaqih MA (2021). Exploring perception and hesitancy toward COVID-19 vaccine: a study from Jordan. Hum Vaccin Immunother.

